# Characterization of negative health outcomes for dialysis events by vascular access type—Tennessee, 2015–2019

**DOI:** 10.1017/ash.2023.310

**Published:** 2023-09-29

**Authors:** Tara Suhs, Alex Kurutz, Christopher Wilson

## Abstract

**Background:** The dialysis patient population is at a higher risk for nosocomial infections as well as related negative consequences including hospitalization and death. The CMS and the state of Tennessee mandate reporting of 3 types of dialysis events: positive blood culture, intravenous antimicrobial starts, and pus, redness, or increased swelling at the access site. We explored hospitalization and death outcomes by vascular access types for dialysis events reported to the NHSN for licensed outpatient hemodialysis clinics in Tennessee from 2015 to 2019. **Methods:** We looked at the frequency of hospitalization and death among those who experienced a dialysis event for 3 types of vascular access: arteriovenous fistula, arteriovenous graft, and tunneled central venous catheter (CVC). Other vascular-access types were excluded due to low usage rates. Odds ratios and confidence intervals were used to quantify the relationship between access type and hospitalization, and access type and death. Pooled analysis was used due to the stable rates of death and hospitalization among access types from 2015 to 2019. **Results:** From 2015 to 2019, 16,742 dialysis events were reported for the 3 access types: 8,055 dialysis events (48.1%) occurred among those with tunneled CVCs, 7,107 (42.5%) occurred among those with fistulas, and 1,580 (9.4%) occurred among those with grafts. Of the 16,742 dialysis events, 3,420 patients (20.4%) were hospitalized either due or related to their dialysis event; 220 (1.3%) deaths occurred either due to or related to the patient’s dialysis event. The odds of being hospitalized was 1.47 (95% CI, 1.29–1.67) times greater in those with grafts compared to those with fistulas. Patients with tunneled CVCs were 1.30 (95% CI, 1.20–1.41) times greater to be hospitalized compared to those with fistulas. The odds of death was 1.09 (95% CI, 0.9–2.5) times greater in those patient with tunneled CVCs compared to those with fistulas, whereas the odds of death among patients with grafts was 0.73 (95% CI, 0.82–1.43) times the odds of death compared to patients with fistulas. **Conclusions:** Overall, our findings conclude hemodialysis patients with tunneled CVCs have an increased risk for the negative health outcomes of hospitalization and death when compared to the other access types, supporting previous studies. Additionally, grafts had a higher risk of hospitalization compared to fistulas, but patients with grafts had lower odds of death than those with fistulas. Further investigation is needed to study how the COVID-19 pandemic may have affected the trends of negative health outcomes related to dialysis events.

**Figure:**
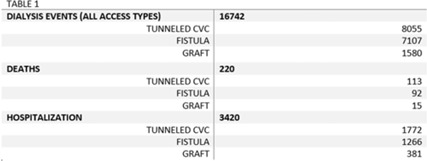

**Disclosures:** None

